# Influence of low amounts of zinc or magnesium substitution on ion release and apatite formation of Bioglass 45S5

**DOI:** 10.1007/s10856-020-06426-1

**Published:** 2020-10-09

**Authors:** R. Wetzel, O. Bartzok, D. S. Brauer

**Affiliations:** Otto Schott Institute of Materials Research, Friedrich Schiller University, Fraunhoferstr. 6, 07743 Jena, Germany

## Abstract

Magnesium and zinc ions play various key roles in the human body, being involved, among others, in skeletal development and wound healing. Zinc is also known to have antimicrobial properties. While low concentrations can stimulate cells in vitro, high concentrations of magnesium or zinc introduced into bioactive glasses significantly reduce glass degradation and ion release and inhibit apatite precipitation. On the other hand, magnesium and zinc ions improve the high temperature processing of bioactive glasses, even when present at low concentrations only. Results here show that by substituting small amounts of Mg or Zn for Ca, ion release remains high enough to allow for apatite precipitation. In addition, magnesium and zinc containing bioactive glasses are shown to be very susceptible to changes in particle size and relative surface area. For a given magnesium or zinc content in the glass, ion release and apatite formation can be enhanced dramatically by reducing the particle size, reaching comparable levels as Bioglass 45S5 of the same particle size range. Taken together, these findings suggest that when introducing these ions into bioactive glasses, ideally low Mg or Zn for Ca substitution as well as small particle sizes are used. This way, bioactive glasses combining good high temperature processing with fast ion release and apatite precipitation can be obtained, providing the potential additional benefit of releasing magnesium or zinc ions in therapeutic concentrations.

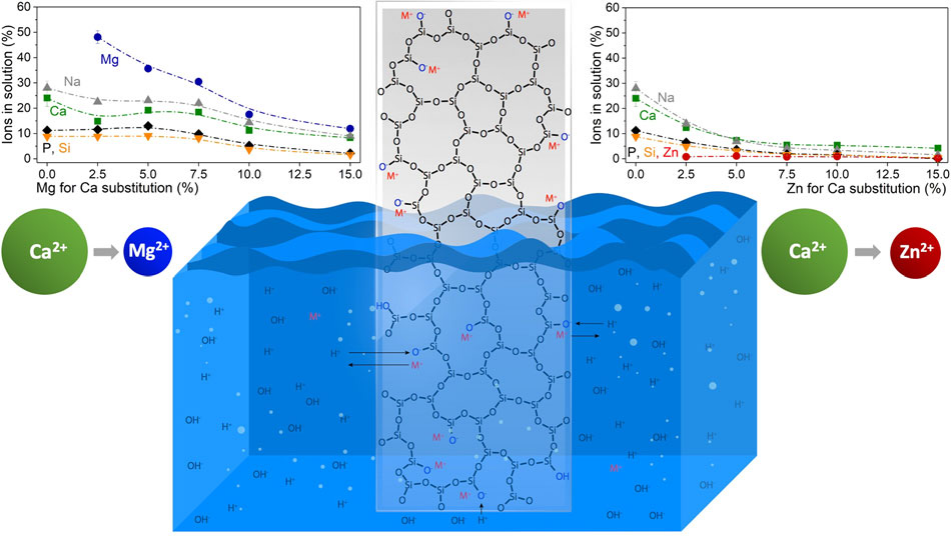

## Introduction

Bioactive glasses are used to regenerate bone or re-mineralise dental tissue [1], and their capacity to release ions is an additional feature besides their degradation and apatite surface mineralisation [2]. This opens possibilities in extending their therapeutic range by incorporating ions which promote a specific physiological response when released into the body [3], and it has been shown that various ions can be successfully incorporated into and released from bioactive glasses. Indeed, we have reported that osteoblasts exposed to early doses of fluoride show increased markers for bone mineralisation in vitro [4] or that lithium ions released from bioactive glasses can upregulate the Wnt pathway in 17IA4 cells in vitro to potentially promote hard tissue repair [5]. Zinc [6] and magnesium [7] have also recently attracted interest as bioactive glass components owing to their key roles in the human body: both ions are known to be co-factors in various enzymes [8–10] and, more importantly, to be important for bone mineralisation and bone density [11, 12].

In a previous study we showed that, despite their similarity in ionic radius and charge [13], Mg^2+^ and Zn^2+^ affect Bioglass degradation, ion release and apatite formation in a very different way, at least when present at high concentrations in the glass [14]. Zinc for calcium substitution caused a dramatic drop in ion release at physiological pH, entirely inhibiting apatite precipitation owing to a lack of available calcium and phosphate ions. By contrast, magnesium acted in a comparable way to calcium, only slightly reducing ion release owing to its higher field strength. Nonetheless, apatite formation was significantly reduced, owing to magnesium ions inhibiting apatite nucleation and crystallisation [15]. As the lowest substitution in our previous study was 25% [14], this raises the question whether Mg or Zn could be incorporated at (lower) levels which do not negatively affect ion release or apatite precipitation, and thus provide the benefit of therapeutic Mg^2+^ or Zn^2+^ release combined with the formation of apatite surface layers. This is particularly interesting as Mg and Zn ions have recently been shown to improve the sintering of bioactive glasses, even at low levels of Mg or Zn for Ca substitution [16]. The aim of the present study was therefore to investigate the effect of low Zn or Mg for Ca substitution on the ion release and apatite precipitation behaviour of Bioglass 45S5 of two different particle size ranges.

## Materials and methods

### Glass synthesis and basic characterisation

Two glass series were prepared based on Bioglass 45S5, where magnesium or zinc were substituted for calcium on a molar basis between 2.5 and 15% (Table 1). Glasses were prepared by a melt-quench route: mixtures of SiO_2_, CaCO_3_, NaPO_3_, MgCO_3_ and ZnO were sintered together in a platinum crucible inside an electric furnace at 1200 °C and then melted for 1 h at 1350 °C. A batch size of ~150 g was used. After melting, the glasses were rapidly quenched into water to prevent crystallisation. Glasses were crushed in a steel mortar, milled in an agate ball mill (KM1, Janetzki; milling time 30 min) and sieved using analytical sieves to obtain coarse (125 ≤ *x* ≤ 250 µm) and fine (≤ 38 µm) glass powder.

**Table 1:** Nominal glass composition (mol%)

	SiO_2_	P_2_O_5_	Na_2_O	CaO	MgO	ZnO
Zn25	46.1	2.6	24.4	20.2	–	6.7
Zn15	46.1	2.6	24.4	22.9	–	4.0
Zn10	46.1	2.6	24.4	24.2	–	2.7
Zn7.5	46.1	2.6	24.4	24.9	–	2.0
Zn5	46.1	2.6	24.4	25.6	–	1.3
Zn2.5	46.1	2.6	24.4	26.2	–	0.7
45S5	46.1	2.6	24.4	26.9	–	–
Mg2.5	46.1	2.6	24.4	26.2	0.7	–
Mg5	46.1	2.6	24.4	25.6	1.3	–
Mg7.5	46.1	2.6	24.4	24.9	2.0	–
Mg10	46.1	2.6	24.4	24.2	2.7	–
Mg15	46.1	2.6	24.4	22.9	4.0	–
Mg25	46.1	2.6	24.4	20.2	6.7	–

Glass monoliths were prepared by re-melting glass frit at 1350 °C, pouring the melt into a brass mould, annealing at 30 K below glass transition temperature and cooling to room temperature in the switched-off furnace overnight. Glass density (*ρ*) was determined on monoliths using helium pycnometry (AccuPyc 1330–1000, Micromeritics GmbH); error is 0.1 g cm^−3^. In addition, molar volume (*V*_m_) of the glass series (and the experimental error) was calculated from glass density as described elsewhere [17]. Structural analysis was performed by powder X-ray diffraction (XRD; Miniflex 300, Rigaku Corporation) and attenuated total reflectance Fourier transform infrared spectroscopy (ATR-FTIR; Cary 630 FTIR, Agilent Technology Inc.).

### Immersion experiments and following characterisation

Immersion experiments were performed in Tris buffer solution (0.062 mol l^−1^), which was prepared by dissolving tris(hydroxymethyl)aminomethane in deionised water and adjusting the pH using HCl solution as described previously [18]. Either coarse or fine glass powder (75 mg) was immersed in 50 ml of Tris buffer solution and stored in a shaking incubator at 37 °C for up to 7 days (6, 12, 24, 72 or 168 h).

At each time point, the pH of the storage medium was measured (pH meter HI 8314 with pH electrode HI 1217 D, Hanna Instruments) and samples were filtered through medium porosity filter paper (5 µm particle retention, VWR International) and acidified using nitric acid (65%). Ion concentrations were determined using inductively coupled plasma optical emission spectrometry (ICP-OES spectrometer 725ES, Agilent). Experiments were performed in triplicate, and results are presented as percentage of ions present in solution relative to the amount of the respective ion present in the untreated glass (mean ± standard deviation). Glass powder samples from each time point were analysed by XRD and ATR-FTIR. For XRD analysis, coarse particles were ball-milled (Pulverisette 23, Fritzsch GmbH; milling time 1 min); for FTIR analysis coarse particles were ground using an agate pestle and mortar.

## Results and discussion

### Density and molar volume

**Fig. 1:**
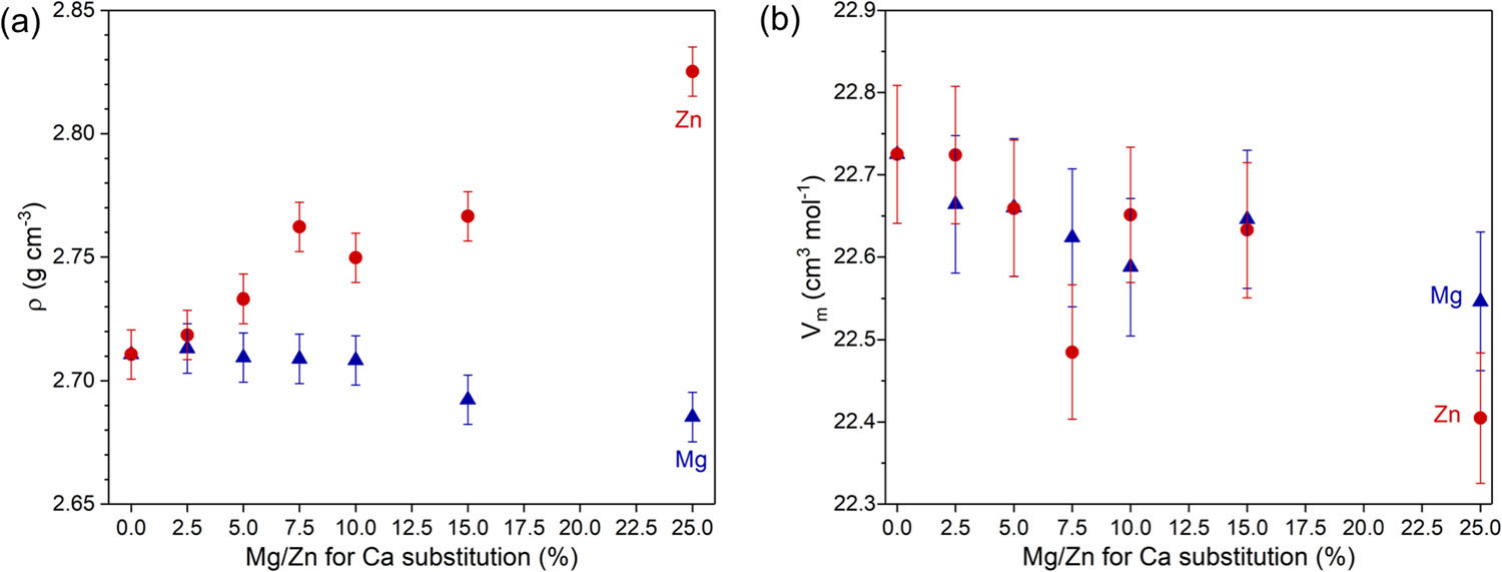
**a** Density and **b** molar volume for Mg (blue triangles) and Zn-substituted (red circles) glasses

Glass monoliths looked clear, and XRD results showed amorphous halos only (not shown), indicating the amorphous structure of the samples. Mg and Zn substitution caused opposite trends in density (Fig. 1a), with glass density decreasing with increasing Mg for Ca substitution, owing to the lower atomic weight of Mg compared to Ca. Zn for Ca substitution caused a density increase, owing to the larger atomic weight of Zn. By contrast, molar volume slightly decreased in both series, which is seen in Fig. 1b despite some scattering of the data. This suggests that the smaller ionic radii of Mg^2+^ (57 pm) and Zn^2+^ (60 pm) compared to Ca^2+^ (100 pm) [13] caused the glass structure to be more compact, similar to observations on lithium for sodium substitution [19].

### pH and ion release

**Fig. 2:**
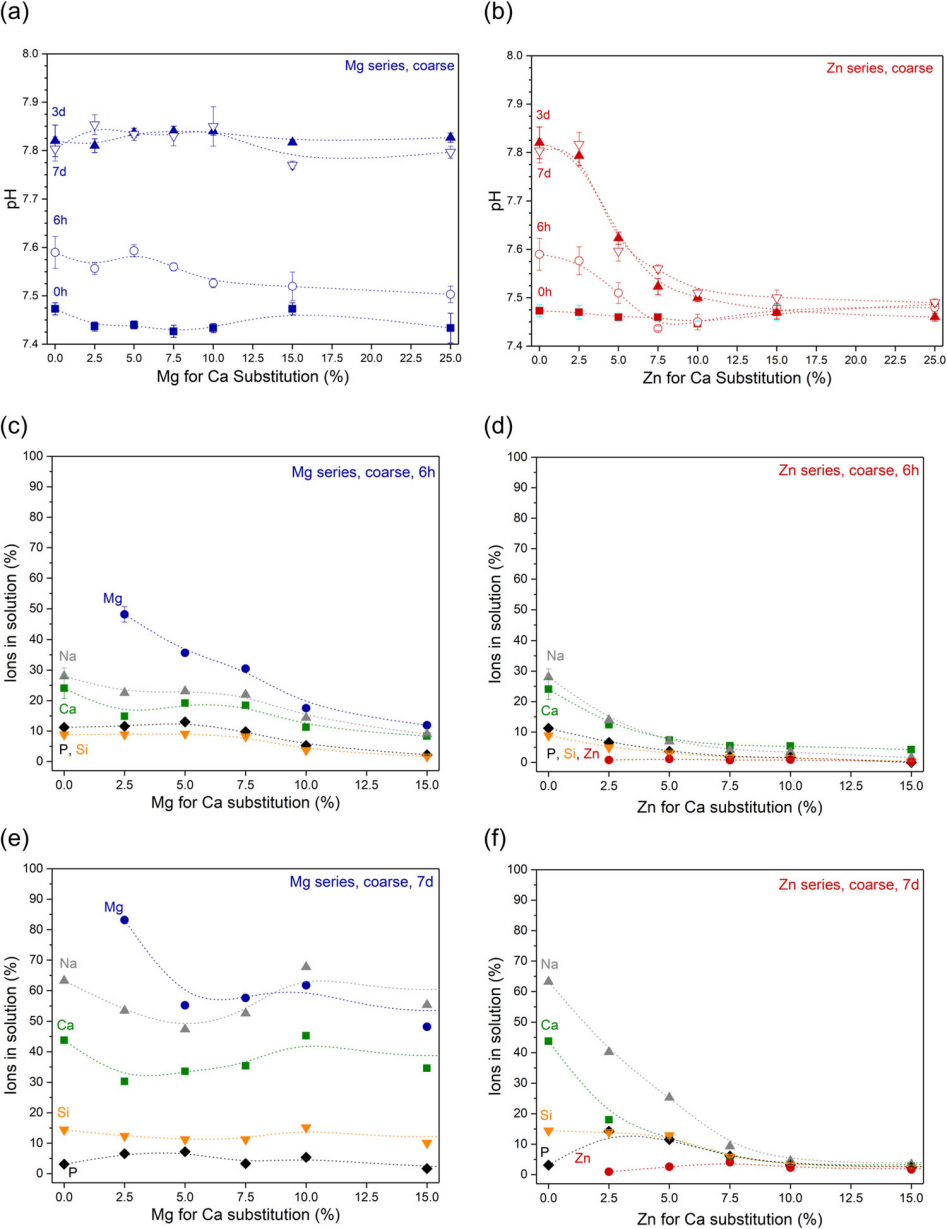
**a**, **b** pH and **c**–**f** normalised ions in solution at (**c**, **d**) 6 h’ and (**e**, **f**) 7 days’ (i.e. 168 h’) immersion of coarse particles of (**a**, **c**, **e**) Mg and (**b**, **d**, **f**) Zn-substituted glasses in Tris buffer solution. (Lines are visual guides only)

When immersed in Tris buffer, all Mg-substituted glasses showed a pH increase over time, which is typical for bioactive glasses [20]; maximum pH was reached within 3 days (Supplementary Fig. S1a). pH trends were comparable to that seen for Bioglass 45S5, and solution pH did not vary pronouncedly with Mg for Ca substitution (Fig. 2a). Of the Zn-substituted glasses, only Zn2.5 showed a comparable trend (Supplementary Fig. S1b), while with increasing Zn for Ca substitution the pH rise was significantly less pronounced (Fig. 2b). Glasses with the highest Zn substitution did not show any pH rise during the duration of the experiment (Supplementary Fig. S1b), in agreement with the results for high substitution compositions (≥25% substitution) presented earlier [21].

The pH increase observed during bioactive silicate glass immersion is known to originate from an ion exchange between modifier cations from the glass and protons from the immersion medium [20, 22]; leaving behind a surplus of hydroxyl ions and, thus, causing the pH to rise [23]. This means that the pH increase can be interpreted as the total of the overall ion exchange having occurred [24]. It is therefore not surprising that the trend observed over time for the relative concentrations of modifier ions in solution (Na, Ca, Mg), shown as a percentage of the ions present in the untreated glass, reflected the trend observed for pH over time (shown for glasses Mg2.5, Mg5, Zn2.5 and Zn5 in Supplementary Fig. S2). When plotting relative concentrations over substitution, concentrations decreased with both Mg and Zn substitution in the glass at early time points (shown for 6 h in Fig. 2c, d). At later time points, by contrast, a clear decrease in concentrations was visible for Zn substitution only, while no clear trend was observed with Mg substitution (shown for 7 days in Fig. 2e, f). This suggests that increasing Mg substitution delayed the ion release from the glass, while Zn substitution, at least above a certain amount, which here was about 7.5%, completely inhibited ion exchange between glass and Tris buffer solution.

The same trend has been observed previously for much higher substitution (25% and above) [21]. Interesting here is that our results show that it is possible to include zinc in bioactive glasses without inhibiting ion release entirely, as long as zinc concentrations in the glass remain below a certain threshold.

Zinc ions are known to play an important role in mammalian systems and to be essential for growth and normal development [25]. They have a stimulatory action on bone formation both in vitro [26] and in vivo [27] and inhibit osteoclastic bone resorption in vitro [28]. While high zinc concentrations have been shown to be cytotoxic in vitro, low concentrations showed stimulatory effects resulting in increased metabolic activity [29]. While absolute zinc concentrations here were low (between 0.001 and 0.006 mM) for all investigated glasses at 6 h of immersion, at later time points zinc concentrations varied with the level of Zn for Ca substitution in the glass, ranging from 0.002 to 0.04 mM at 7 days (Supplementary Fig. S1d).

For magnesium, changes in ion release behaviour with substitution seem to originate from differences in field strength (or charge-to-size ratio) [13] only, as the larger charge-to-size ratio of Mg^2+^ compared to Ca^2+^ results in stronger ionic bridges between non-bridging oxygen (NBO) atoms. Mg^2+^ is typically being present in lower coordination numbers than calcium, with molecular dynamics simulations giving an average coordination number of 5 for magnesium compared to 6 for calcium in substituted Bioglass 45S5 [30]. While this might be expected to weaken the overall bond strength in Mg-substituted glasses, the higher average field strength seems to compensate for the decrease in coordination number, making the glass more stable against water attack and ion release. Another factor to consider here is the more compact packing of the ions in the glass, as indicated by the smaller molar volume shown in Fig. 1. If the glass components are packed closer to one another, it becomes more difficult for water molecules to penetrate the network [22] and allow for ion exchange to occur.

Relative Mg concentrations in the glass at 6 h (Fig. 2c) decreased with increasing Mg for Ca substitution, suggesting that with increasing Mg concentration in the glass, a lower percentage of the Mg ions was released into the surrounding medium. At 7 days, this trend was much less noticeable (Fig. 2e). Absolute Mg concentrations (Supplementary Fig. S1c) confirm this, showing relatively constant Mg concentrations around 0.1 mM at 6 h, while concentrations at 7 days increased nearly linearly with Mg for Ca substitution.

**Fig. 3:**
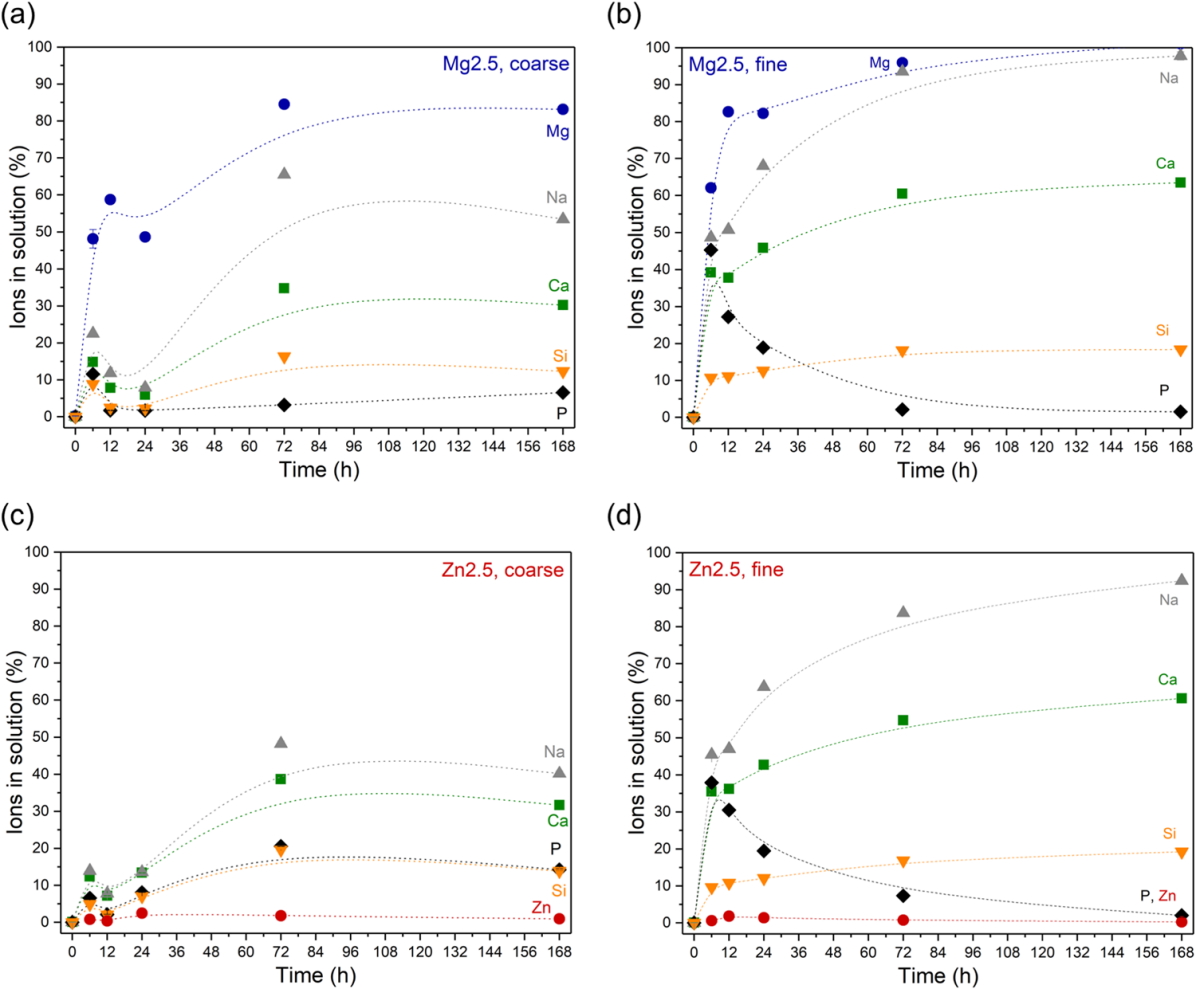
Comparison of normalised ion concentrations in solution after immersion of (**a**, **c**) coarse and (**b**, **d**) fine particles of glasses (**a**, **b**) Mg2.5 and (**c**, **d**) Zn2.5 in Tris buffer solution over time. (Lines are visual guides only)

Figure 3 compares relative ion concentrations in Tris buffer solution during immersion of coarse and fine glass powder of glasses Mg2.5 or Zn2.5. Results show that a reduction in particle size and, thus, an increase in relative surface area resulted in an increase in relative (and, not shown, absolute) ion concentrations in solution. This is not surprising, as the underlying ion exchange mechanism occurs at the glass/water interface, and an increase in relative surface area is therefore expected to allow for faster ion exchange to occur [31]. This was confirmed by fine glass powder showing a more pronounced pH rise than coarse glass powder (Supplementary Fig. S3a).

pH trends for glasses 45S5, Mg2.5 and Zn2.5 were comparable when using the same particle size range (Supplementary Fig. S3a). By contrast, reducing the particle size seemed to affect ion release more for glass Zn2.5 than for Mg2.5 (Fig. 3). This difference cannot easily be explained by differences in cation charge-to-size ratio, as Mg^2+^ and Zn^2+^ have nearly identical ionic radii [13]. In our previous publications on Zn containing bioactive glasses [21, 32], we reasoned that Zn acted like an intermediate ion in bioactive glasses by (partially or fully) entering the silicate network, similar to the behaviour of aluminium in aluminosilicate glasses. This would result in Zn ions being “locked into” the glass network at neutral to alkaline pH but being released quickly at acidic pH as shown for Zn-substituted Bioglass 45S5 [21]. However, this does not explain why a change in relative surface area affected Zn-substituted glasses much more than Mg substituted ones. To fully explain these differences, detailed structural analyses of the role of Mg and Zn ions in the glass network may be necessary.

The results shown here suggest that changing the particle size is an efficient means of tailoring the ion release particularly for Zn containing bioactive glasses. Figure 3b, d confirms that the ion release profiles of fine powders of glasses Mg2.5 or Zn2.5 look like the well-known ion release profiles of Bioglass 45S5 (Supplementary Fig. S3b) [24], suggesting that these two glasses, at least when used as fine powders, may behave in a similar way to 45S5, with the added benefits of Mg or Zn ion release [33] and improved sintering [16].

### XRD and FTIR

**Fig. 4:**
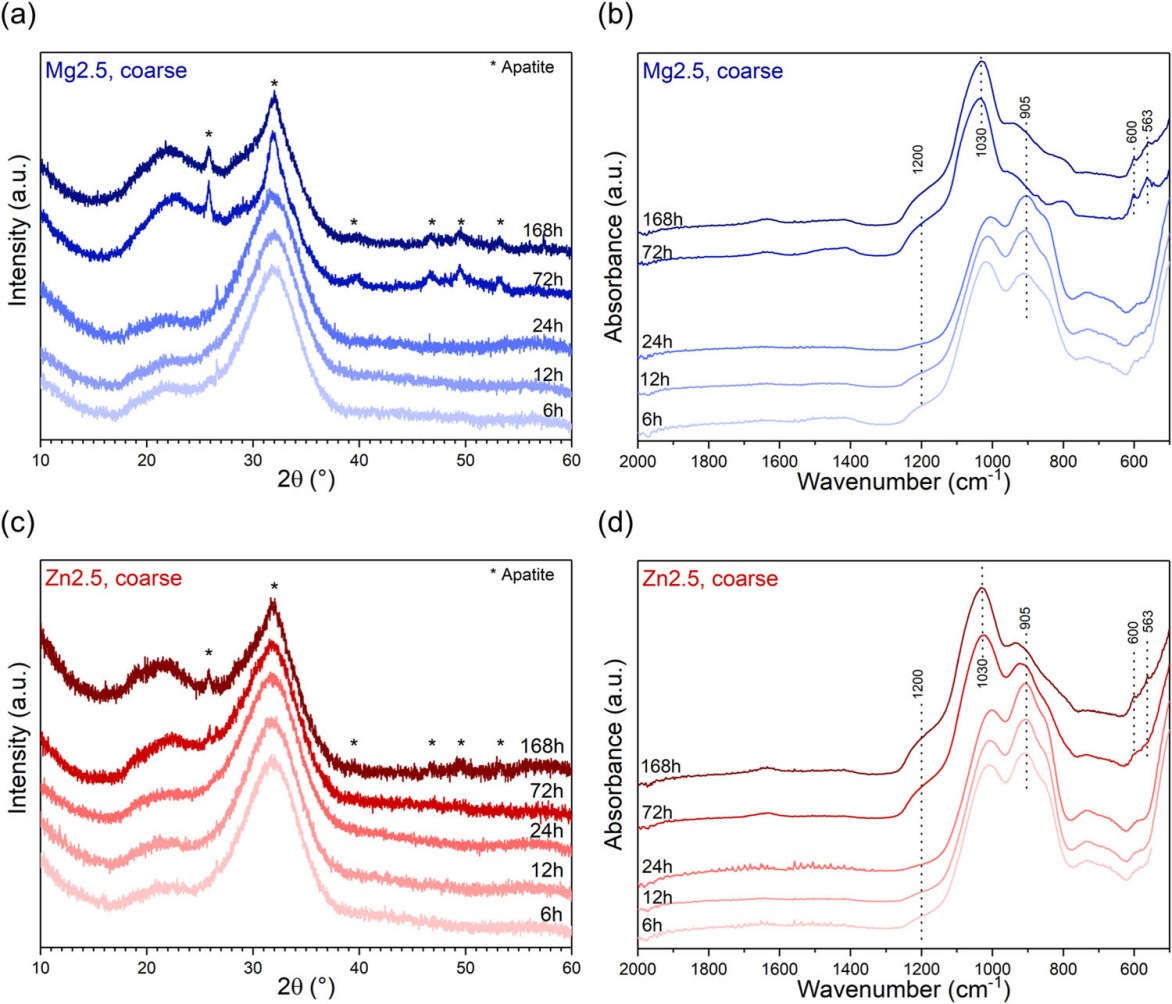
**a**, **c** XRD patterns and **b**, **d** ATR-FTIR spectra of coarse particles of glasses (**a**, **b**) Mg2.5 and (**c**, **d**) Zn2.5 after immersion in Tris buffer solution for various time periods

Figure 4 shows the XRD patterns and FTIR spectra of coarse particles of glasses Mg2.5 and Zn2.5 at various time points of immersion in Tris buffer solution. Up to 24 h, no pronounced changes are observed, suggesting minimal reaction with the surrounding medium. FTIR spectra of bioactive glasses typically show fast disappearance of the NBO-related bands (maxima at about 850–900 cm^−1^), owing to the above-mentioned ion exchange of modifier ions from the glass, connected ionically to NBO, for protons from the surrounding aqueous medium [24]. Here, no significant decrease in the intensity of these bands was observed for the first 24 h of the immersion studies. For glass Zn2.5 this was to be expected, as only a minor percentage of modifier ions was found in solution at this time point (Fig. 3c). For glass Mg2.5, by contrast, about 60% of magnesium ions in addition to about 10% each of sodium and calcium ions were found in solution at 12 h (Fig. 3a), and this should be reflected in a decrease in the intensity of the NBO band, especially as FTIR measurements are quite sensitive to surface features. However, grinding of coarse particles before FTIR analysis may have affected these results, as fresh surfaces were created during the grinding process, which are likely to have shown the features of the unreacted glass in addition to the reacted surfaces.

The ion exchange occurring between glass and immersion medium can also be reflected in XRD patterns through a shift in the position of the amorphous halo from about 32 to 22°2*θ*, caused by the formation of an ion-depleted glass, often referred to as a silica gel [34]. XRD patterns here (Fig. 4a, c) show a weak amorphous halo at about 22°2*θ* in addition to the one at 32°2*θ*, which may originate from silica gel formation as early as 6 h, even for glass Zn2.5.

At later time points of immersion (72 and 168 h), the intensity of the NBO-related FTIR bands had decreased pronouncedly for both glasses (Fig. 4b, d). This decrease was even more dramatic for glass Mg2.5 than for Zn2.5, in agreement with higher percentages of ions found in solution for Mg2.5. In addition, the typical split band at 563 and 600 cm^−1^ was present for glass Mg2.5 from 72 h and for Zn2.6 at 168 h, suggesting apatite formation, in agreement with a sharp phosphate band at about 1030 cm^−1^ [34].

The onset of apatite formation often coincides with a decrease in phosphate concentration in solution [20, 31], at least when performing immersion experiments in media which originally did not contain any phosphate, such as Tris buffer solution. All phosphate consumed during apatite precipitation needs to be released from the glass into the immersion medium before any apatite formation can occur. As typical bioactive glasses contain and, thus, release much more calcium than phosphate, phosphate is the limiting factor for apatite formation, and no pronounced decrease in calcium concentrations is typically observed. Here, phosphate concentrations for Mg2.5 (Fig. 3a) had decreased at 12 h already, i.e. at a time point much earlier than suggested by XRD and FTIR results. This could possibly be explained by an amorphous calcium phosphate (CaP) having formed first, rather than apatite (or octacalcium phosphate [35]). Additionally, the creation of fresh surfaces during grinding or milling before FTIR and XRD analyses may be the reason why certain features of the reacted surfaces were not detected. Zn2.5 did not show any pronounced decrease in phosphate concentrations in solution over the duration of the immersion study (Fig. 3c), in agreement with weakly pronounced apatite features in XRD and FTIR at 168 h.

Apatite formation was confirmed by XRD patterns showing the typical apatite-related reflections (Fig. 4a, c) at the same time points. As we did not investigate any time points between 72 and 168 h, we cannot make any statements about the exact lag between the onset of apatite formation for Mg2.5 and Zn2.5, but the presence of the phosphate FTIR band at 1030 cm^−1^ at 72 h for Zn2.5 indicates that apatite formation had already started by this time point. It becomes clear, however, that the two glasses differ much less in their apatite forming capacity than Mg and Zn containing glasses of higher substitution [21].

**Fig. 5:**
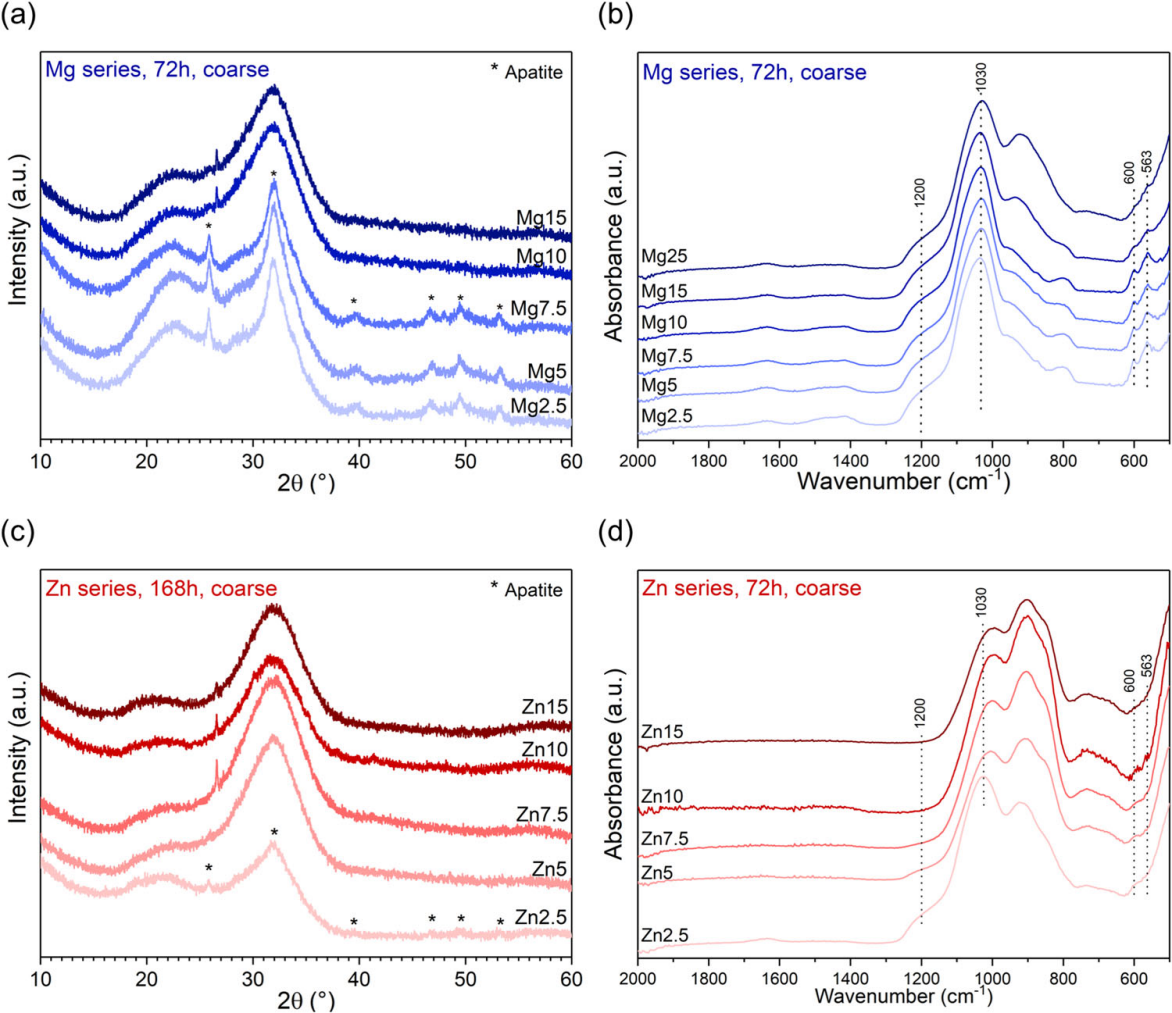
**a**, **c** XRD patterns and **b**, **d** ATR-FTIR spectra of coarse particles of (**a**, **b**) Mg and (**c**, **d**) Zn-substituted glasses after immersion in Tris buffer solution for (**a**, **b**, **d**) 3 days (72 h) or (**c**) 7 days (168 h)

Figure 5 clearly illustrates that the time point of silica gel formation or apatite precipitation varies with of Mg or Zn for Ca substitution, agreeing with the results of the ion release studies. These findings are particularly interesting for Zn-substituted glasses, as in our previous study, none of the Zn-substituted glasses (≥25% Zn for Ca substitution [21]) showed any changes in their FTIR spectra with immersion time in Tris buffer. FTIR spectra of glasses Zn2.5 to Zn7.5 at 72 h of immersion might possibly show some weak features at 560–600 cm^−1^, indicating formation of an amorphous calcium phosphate (CaP). By contrast, XRD patterns at 168 h do not show significant differences for substitutions of 5% and above, except for a small reflex at 26.6°2*θ* for glasses Zn7.5 to Zn15, which could not be assigned to any crystal phases. In Supplementary Fig. S3c, the FTIR spectrum of untreated glass Zn15 together with those of Zn15 at up to 168 h of immersion are plotted on top of one another, showing that no structural changes could be detected by FTIR over 1 week of immersion. This shows that the glasses with high Zn for Ca substitution are surprisingly stable in an aqueous environment and should not really be termed “bioactive” at all.

Glasses in the Mg series show more continuous changes with substitution. At 72 h, the intensity of the NBO-related bands in FTIR spectra (Fig. 5b) increased with increasing substitution, suggesting less ion exchange with increasing substitution. This is interesting, as pH and ion concentrations in solution (Fig. 2a, c) showed no such trend. The intensity of the phosphate-related bands (563, 600 and 1030 cm^−1^) decreased with increasing Mg substitution, suggesting a decreasing tendency to precipitate apatite. This was confirmed by XRD patterns, which at 72 h showed reflexes corresponding to apatite for glasses Mg2.5 to Mg7.5 only.

FTIR of untreated fine powder 45S5 (Supplementary Fig. S4b) and possible also those of some of the glasses with lowest substitution (Fig. 2b, d) show a low intensity feature at about 580–600 cm^−1^. A broad band in this region is often taken as an indication of formation of an amorphous CaP layer [36], and it may have been caused by reaction with air humidity [37], despite storage in a desiccator.

**Fig. 6:**
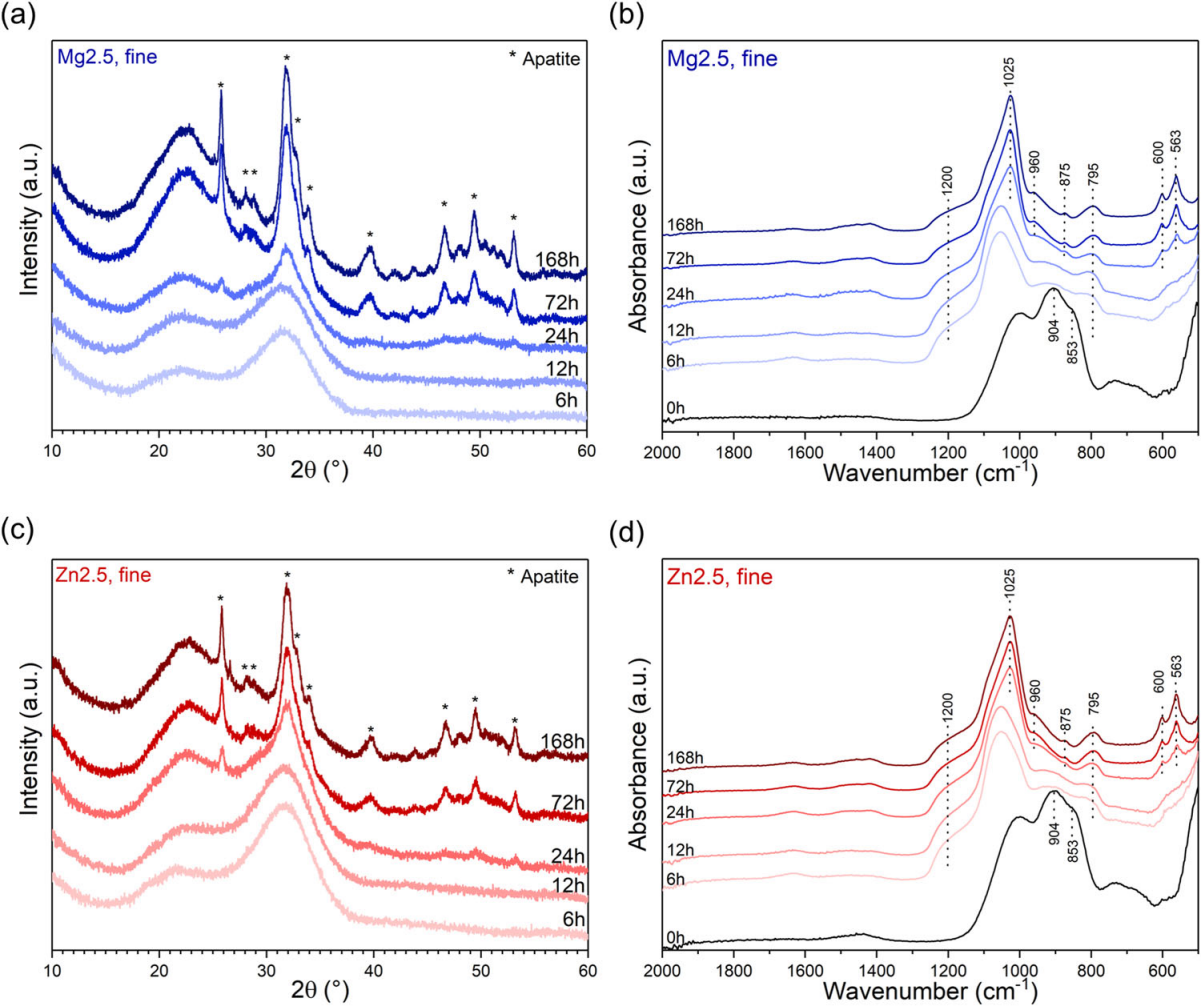
**a**, **c** XRD patterns and **b**, **d** ATR-FTIR spectra of fine particles of (**a**, **b**) Mg2.5 and (**c**, **d**) Zn2.5 after immersion in Tris buffer solution for various time periods

FTIR spectra of fine powder of glasses Mg2.5 and Zn2.5 (Fig. 6b, d) show nearly complete disappearance of the NBO-related bands at 6 h, suggesting much faster ion exchange for smaller particles (and larger relative surface areas) than for larger ones. This is in excellent agreement with pH results (Supplementary Fig. S3a) and ions in solution (Fig. 3) discussed above. Apatite-related features in XRD patterns or FTIR spectra also appeared much faster for fine powder (at 24 h) than for coarse one (72 and 168 h for Mg2.5 and Zn2.5, respectively). This suggests that the concentrations of magnesium or zinc ions present in solution for these glasses were not sufficient to significantly inhibit apatite precipitation.

The most interesting aspect is that the differences in apatite formation between fine powders of Mg2.5 and Zn2.5 are negligible, unlike those for coarse powders. XRD and FTIR results of fine powders of Mg2.5 and Zn2.5 are also very similar to those of 45S5 (Supplementary Fig. S4). This agrees perfectly with the findings for ion release discussed above, and it suggests that by using smaller particle size ranges, bioactive glasses containing small amounts of magnesium or zinc ions form apatite about as quickly as 45S5 does, while providing the release of magnesium or zinc ions in addition to calcium, phosphate or silicon.

## Conclusion

Despite their similarity in size and charge, magnesium and zinc ions affect the in vitro degradation behaviour of bioactive glasses in very different ways. Zinc has a pronounced effect on ion release, possibly by stabilising the silicate network through its character as an intermediate ion. With increasing substitution, the release of zinc ions and silica species dropped to nearly zero. This was in pronounced contrast to magnesium, which had a lesser effect on ion release and showed relatively large percentages of magnesium (up to 80%) to be released within a few hours.

Here, we show that by keeping magnesium or zinc concentrations low—but in the range previously shown to improve bioactive glass sintering [16]—overall ion release can be enhanced and apatite formation improved. Ion release and apatite formation can be increased dramatically for a given magnesium or zinc content in the glass by reducing the glass particle size. Fine glass particles react about as quickly as 45S5 particles of the same size, while additionally providing the release of magnesium or zinc ions in potentially therapeutic concentrations.

## Supplementary information

Supplementary Figures

>

Raw Data

## Data Availability

Raw data were submitted as an MS Excel file.
