# Supplementary material for: Influence of low amounts of zinc or magnesium substitution on ion release and apatite formation of Bioglass 45S5
Source: J Mater Sci Mater Med. 2020 Oct 9;31(10):86. doi: 10.1007/s10856-020-06426-1 (PMC7547032; doi:10.1007/s10856-020-06426-1)
Supplement: Supplementary file 1 — Supplementary Figures [file 10856_2020_6426_MOESM1_ESM.pdf]

Supplementary Figures

Influence of low amounts of zinc or magnesium substitution on ion release and apatite formation of Bioglass 45S5

R. Wetzel, O. Bartzok, D.S. Brauer*

Otto Schott Institute of Materials Research, Friedrich Schiller University, Fraunhoferstr. 6, 07743 Jena, Germany

*delia.brauer@uni-jena.de

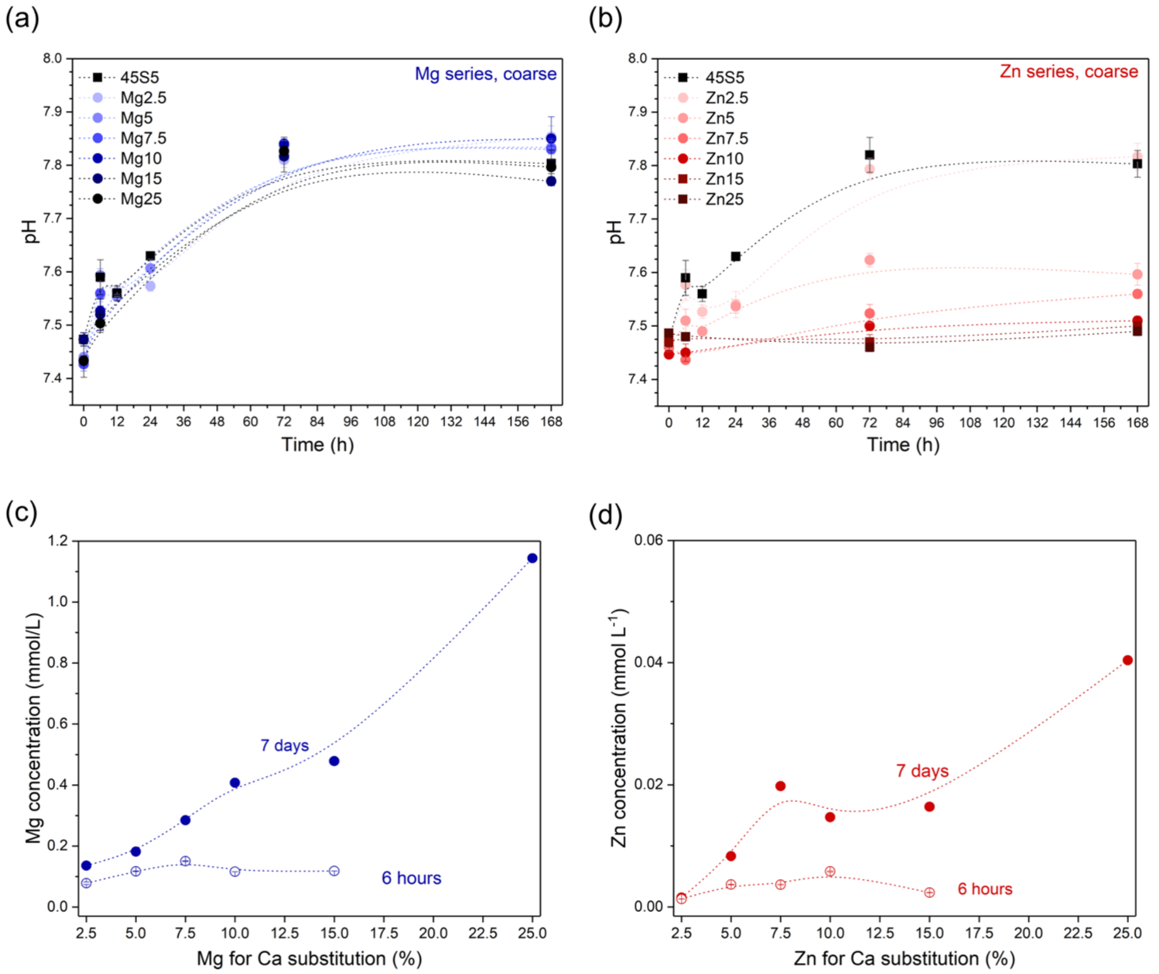

Supplementary Figure S1: (a,b) pH of Tris buffer solution after immersion of coarse glass particles of (a) Mg and (b) Zn substituted glasses over time. (c,d) Absolute (c) magnesium and (d) zinc concentrations in Tris buffer solution during immersion of coarse glass particles.

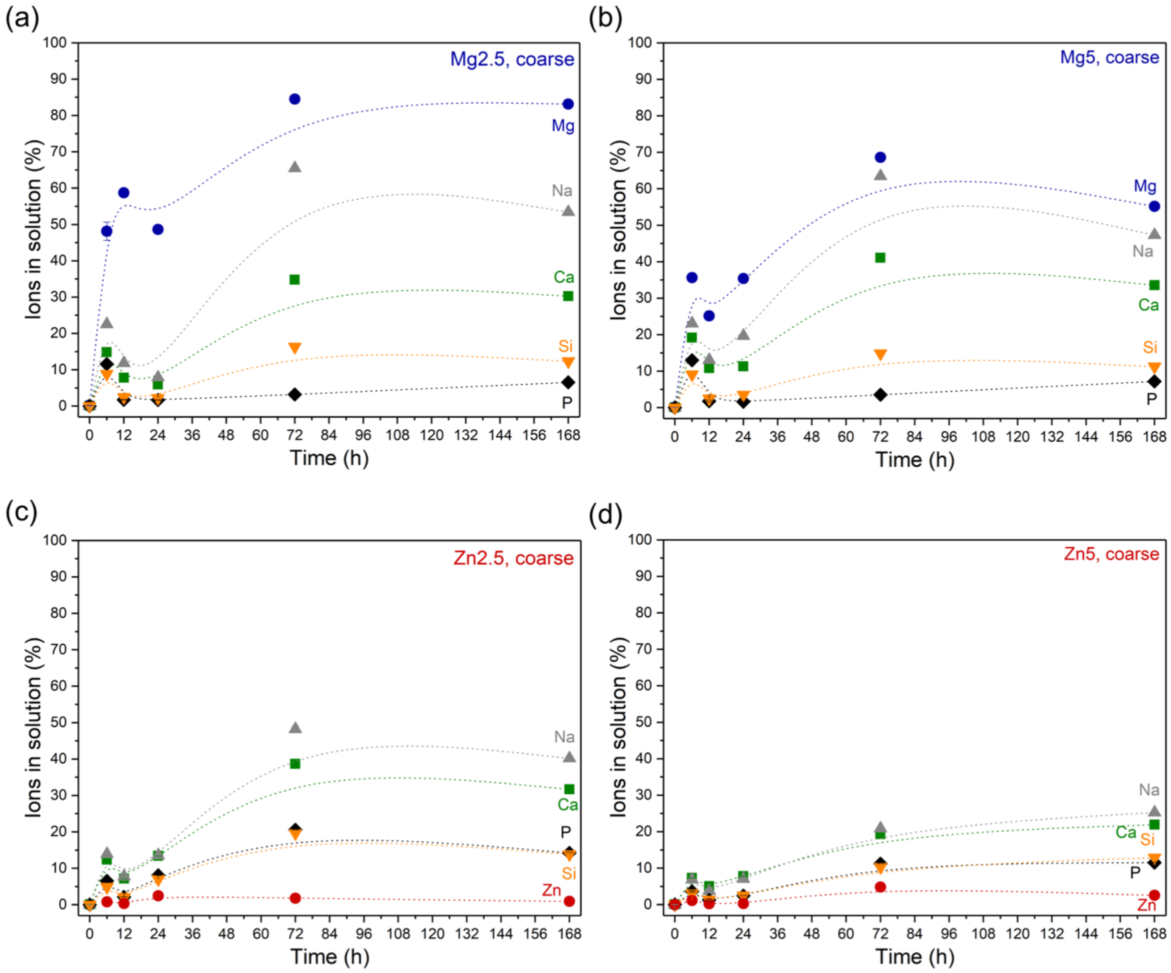

Supplementary Figure S2: Normalised ion concentrations in Tris buffer solution during immersion of coarse glass particles of (a) Mg2.5, (b) Mg5, (c) Zn2.5 and (d) Zn5.

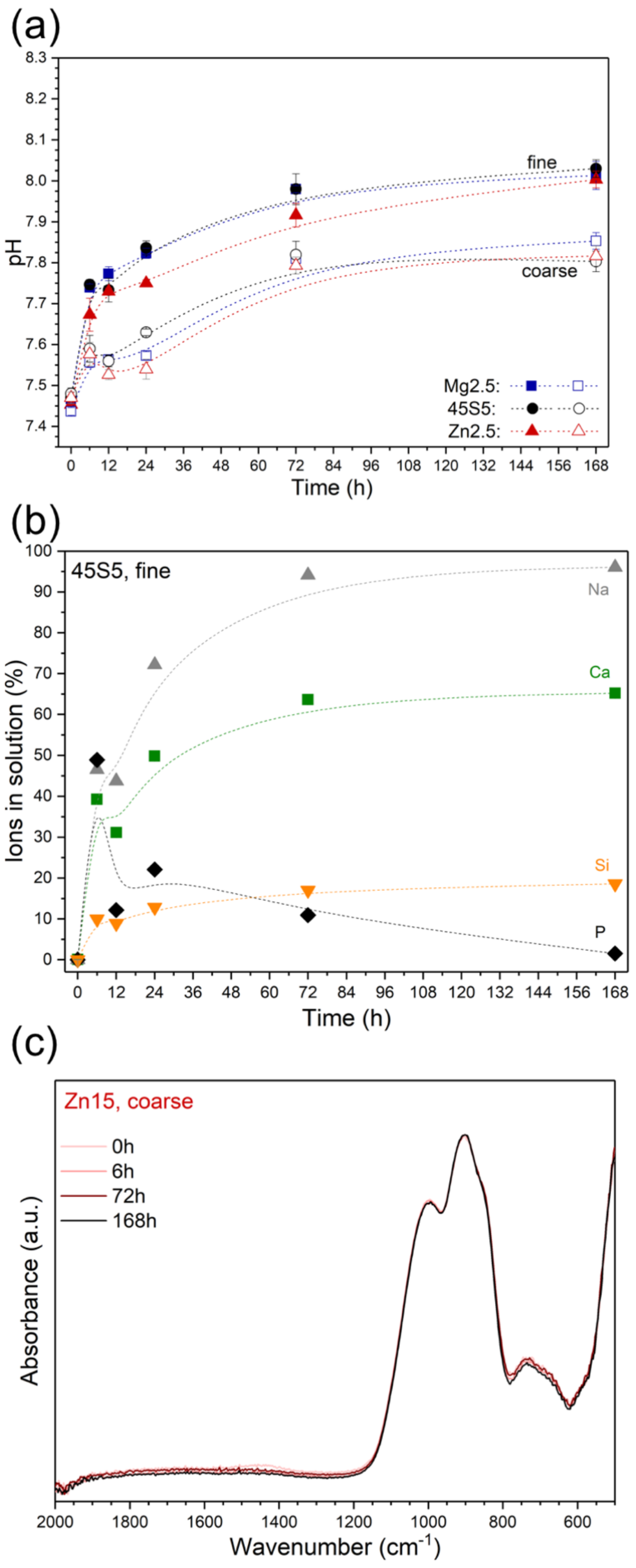

Supplementary Figure S3: (a) pH of Tris buffer solution over time during immersion of fine or coarse particles of glasses Mg2.5, 45S5 or Zn2.5. (b) Normalised ion concentrations over time during immersion of fine particles of 45S5. (c) ATR-FTIR spectra of glass Zn15 (coarse particles) immersed in Tris buffer solution for up to 7 days.

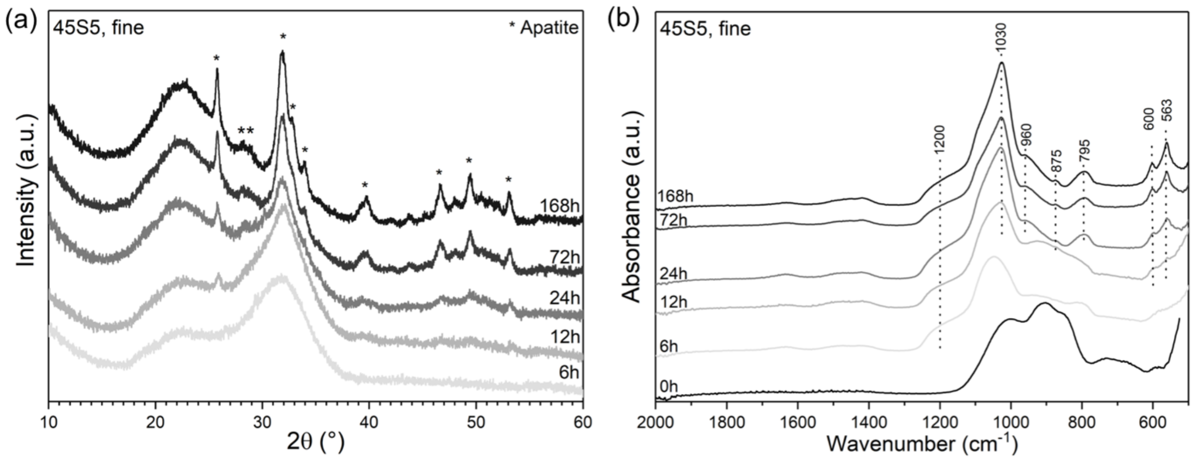

Supplementary Figure S4: (a) XRD patterns and (b) ATR-FTIR spectra of fine particles of 45S5 after immersion in Tris buffer solution for various time periods.
